# Smartphone-based dual radiometric fluorescence and white-light imager for quantification of protoporphyrin IX in skin

**DOI:** 10.1117/1.JBO.28.8.086003

**Published:** 2023-08-26

**Authors:** Alberto J. Ruiz, Richard Allen, Mia K. Giallorenzi, Kimberley S. Samkoe, M. Shane Chapman, Brian W. Pogue

**Affiliations:** a Dartmouth College, Thayer School of Engineering, Hanover, New Hampshire, United States; b QUEL Imaging, LLC, White River Junction, Vermont, United States; c Dartmouth Health, Department of Dermatology, Lebanon, New Hampshire, United States; d University of Wisconsin–Madison, Department of Medical Physics, Madison, Wisconsin, United States

**Keywords:** fluorescence imaging, smartphone, protoporphyrin IX, actinic keratosis, treatment planning, radiometric imaging

## Abstract

**Significance:**

The quantification of protoporphyrin IX (PpIX) in skin can be used to study photodynamic therapy (PDT) treatments, understand porphyrin mechanisms, and enhance preoperative mapping of non-melanoma skin cancers.

**Aim:**

We aim to develop a smartphone-based imager for performing simultaneous radiometric fluorescence (FL) and white light (WL) imaging to study the baseline levels, accumulation, and photobleaching of PpIX in skin.

**Approach:**

A smartphone-based dual FL and WL imager (sDUO) is introduced alongside new radiometric calibration methods for providing SI-units of measurements in both pre-clinical and clinical settings. These radiometric measurements and corresponding PpIX concentration estimations are applied to clinical measurements to understand mechanistic differences between PDT treatments, accumulation differences between normal tissue and actinic keratosis lesions, and the correlation of photosensitizer concentrations to treatment outcomes.

**Results:**

The sDUO alongside the developed methods provided radiometric FL measurements ($\mathrm{nW}/{\mathrm{cm}}^{2}$) with a demonstrated sub nanomolar PpIX sensitivity in 1% intralipid phantoms. Patients undergoing PDT treatment of actinic keratosis (AK) lesions were imaged, capturing the increase and subsequent decrease in FL associated with the incubation and irradiation timepoints of lamp-based PDT. Furthermore, the clinical measurements showed mechanistic differences in new daylight-based treatment modalities alongside the selective accumulation of PpIX within AK lesions. The use of the radiometric calibration enabled the reporting of detected PpIX FL in units of $\mathrm{nW}/{\mathrm{cm}}^{2}$ with the use of liquid phantom measurements allowing for the estimation of *in-vivo* molar concentrations of skin PpIX.

**Conclusions:**

The phantom, pre-clinical, and clinical measurements demonstrated the capability of the sDUO to provide quantitative measurements of PpIX FL. The results demonstrate the use of the sDUO for the quantification of PpIX accumulation and photobleaching in a clinical setting, with implications for improving the diagnosis and treatment of various skin conditions.

## 1 Introduction

The detection and quantification of protoporphyrin IX (PpIX) is an important diagnostic tool in various medical fields, including dermatology and oncology.^1^^–^^3^ Within dermatology, PpIX-based skin photodynamic therapy (PDT) is increasingly used for the clinical treatment of actinic keratosis (AK) lesions, replacing traditional cryosurgery, chemical peel, and curettage treatments.^4^^,^^5^ Despite the widespread clinical use of skin PDT, the primary treatment outcome of AK lesion clearance rates varies widely, which is primarily caused by heterogeneous accumulation of the photosensitizer PpIX between patients and between lesions.^6^^,^^7^ Furthermore, the presence and accumulation of PpIX in skin is also related to conditions like porphyria^8^^,^^9^ and can be used for the preoperative mapping of non-melanoma skin cancers.^10^^,^^11^ Therefore, the ability to visualize and quantify skin PpIX in clinical settings can be used to help guide critical decisions at the point-of-care, including mitigative actions to improve patient outcomes. Imaging and spectroscopy techniques have shown potential for PpIX quantification, with fluorescence (FL) imaging techniques demonstrating great promise for non-invasive measurement of PpIX in the skin.^6^^,^^12^ Additionally, smartphone-based imaging (SBI) devices have emerged as a promising approach for PpIX quantification, offering cost-effective solutions that could be integrated into the clinical workflow.^13^^–^^15^

Generally, SBI devices for quantitative applications require the control of the image acquisition settings (exposure, ISO, and focal distance) and access to the RAW pixel data to provide a full system characterization. This system characterization includes the pixel spectral response, flat field image, image noise, and complete photon transfer curve.^16^ Using smartphone system characterization, image acquisition setting control, and RAW pixel data eliminates “black-box” processing and can decouple imaging results from individual phone performance to allow for intersystem comparisons and facilitate translation into the clinical setting.^17^^,^^18^ Complementing the RAW pixel acquisition with the control of image capture settings, in-phone image analysis, and customized user interfaces can be used to facilitate the translation of smartphone-based systems within existing clinical workflows.^17^

In this study, we present an SBI capable of performing simultaneous radiometric FL and white light (WL) imaging to study the baseline levels, accumulation, and photobleaching of PpIX in skin. The smartphone-based dual FL and WL imager (sDUO) uses the dual-camera imaging module of the iPhone 11, alongside optical components and custom electronics, to simultaneously capture both PpIX FL and skin WL images in a “single-click” acquisition. The study discusses the full system characterization and radiometric calibration alongside liquid phantom, pre-clinical, and clinical measurements. The presented clinical measurements focus on the use of the sDUO for the assessment of mechanistic differences between lamp-based and indoor daylight PDT treatments of AK lesions.

## 2 Methods

### 2.1 sDUO Overview and Components

**Fig. 1:**
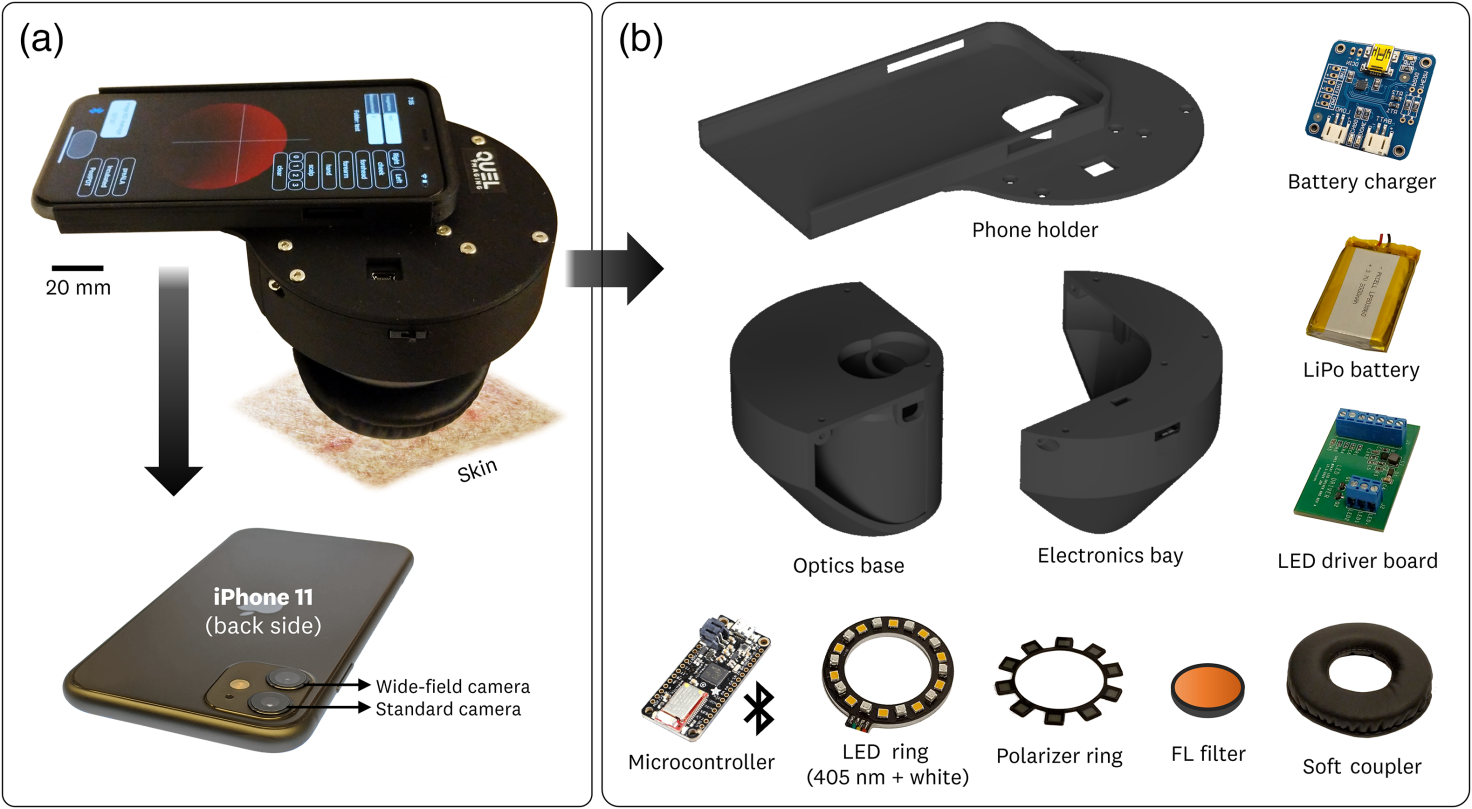
sDUO component overview. (a) The assembled imager uses an iPhone 11 and its standard and wide-field cameras alongside custom software to sequentially capture the FL and white-light images. (b) The electric and optical components are integrated within the 3D-printed optics base and electronics bay. The components are scaled individually for visual clarity.

The assembled sDUO is shown in Fig. 1(a), where the iPhone 11’s standard and wide-field cameras are used for each respective imaging modality. The sDUO integrates eight components within its 3D printed housing. The housing is composed of the phone holder, optics base, and electronics bay [Fig. 1(b)].

The system is powered using a LiPo battery with a battery charger managing the delivery of power to the microcontroller and light emitting diode (LED) driver board. The LED driver board operates the custom LED ring (composed of 405 nm and white-light LEDs) using a constant current. The microcontroller communicates via Bluetooth with the iPhone 11 to coordinate the LED ring activation with the image acquisition. A polarizer ring is used on the white LEDs, with a corresponding cross-polarizer on the wide-field camera, to eliminate reflections from the imaging plane. A long-pass dielectric filter is placed at the aperture of the standard camera to isolate the PpIX FL signal. A cushioned soft ring is used at the aperture of the optics base to couple the device to the skin and create an isolated lighting environment. Each of the components alongside the custom iOS application is discussed in detail in the sections below. Some of the presented components, including the LED ring, soft coupler, and battery charger, are inherited from the first iteration of the smartphone-based device that was published in a previous study.^15^^,^^19^

#### 2.1.1 Smartphone and iOS application

The system design uses an iPhone 11 (Model MWKU2LL/A, Apple, Cupertino, United States) running iOS 14 software. The 12 MP standard back-facing camera (f/1.8) was used for FL imaging given its ability to manually control camera parameters and provide the RAW 12-bit pixel intensity values. The ultrawide 12MP camera (f/2.4 120 deg field-of-view) was used as the WL image detector. The ultrawide camera does not support RAW images, so the generated WL images undergo the standard iOS image processing, resulting in an 8-bit RGB image.

A native iOS application was developed to fix imaging parameters, acquire images from both cameras, generate RAW output images, access RAW pixel values for in-phone processing, and organize and label imaging data. The application was developed in the Swift programming language using Xcode 12. Within this coding environment, the AVCaptureSession and AVCapturePhotoSettings classes were used to control the image acquisition parameters, including the image exposure duration, ISO level, image focus distance, image file type (RAW versus iOS processed), and white balance for the WL images. Access to the RAW pixel buffer was also achieved, which enabled in-phone processing of images, including real-time reporting of average FL intensity values for captured images.

**Fig. 2:**
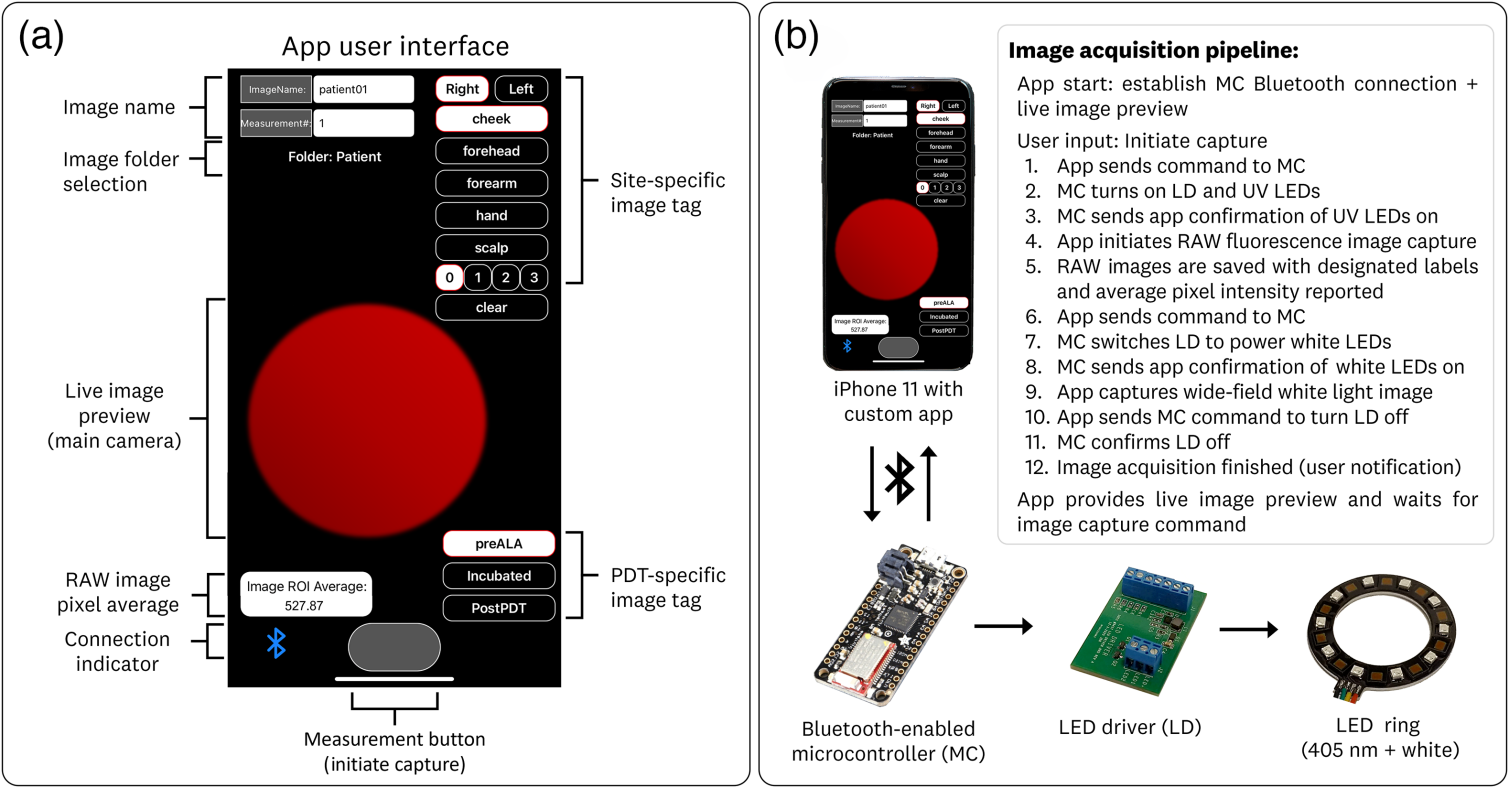
Overview of the (a) custom app interface and (b) image acquisition pipeline. The app user interface allows for “single click” image acquisition with fixed imaging parameters and facilitates the organization, naming, and labeling of acquired images. The image acquisition pipeline uses two-way communication between the application and microcontroller to synchronize the 405 nm and white-light LEDs for their respective image acquisition.

A custom user interface was developed to enable “single-click” image acquisition, provide real-time FL signal levels, and facilitate the labeling of images within the clinical environment (Fig. 2). The app interface [Fig. 2(a)] uses a single button (“measurement button”) for the sequential acquisition of the FL and white-light images. Upon an image acquisition, the app interface provides the average RAW pixel values for a 100×100  pixel region-of-interest (ROI) centered on the FL image. The imaging and analysis pipeline developed allows for the implementation of custom image processing algorithms for in-phone image processing.

The image acquisition parameters [exposure time, ISO, focus distance] were fixed for the standard camera [0.1 s, 400 ISO, 0.0 (closest)] to provide repeatable FL imaging measurements using the iOS RAW imaging pipeline. The 0.1 s exposure time was identified as the largest feasible collection that did not introduce noticeable motion artifacts from the handheld operation of the device. The white-balance of the wide-field camera was fixed to 3000 k to provide consistent iOS processing of the WL images.

A Bluetooth indicator provides the user with verification that the application is connected to the microcontroller to initiate the image acquisition process, which is outlined in Fig. 2(b). Briefly, the process involves two-way communication between the application and the microcontroller, which controls the LED driver output to turn on/off the independent LED ring channels (405 nm and WL). The total time for the dual image acquisition is <2  s, resulting in the generation of three images: a RAW FL image (.DNG file type), a red-pixel only RAW FL image (.DGN file type), and a WL RGB image (.PNG file type). The red-pixel only RAW FL image is used as the PpIX signal image because it provides further optical specificity by isolating the photosensitizer FL while rejecting the excitation wavelengths. The generated RAW images contain 12-bit data within a 16-bit container. The RGB images provide 8-bit data for each red, green, and blue channel (24-bit color).

Furthermore, the app allows for the labeling of images and the creation of folders for the patient and sample-specific image storage. Additionally, the custom creation of “image tags” for the streamlined categorization of acquired data was also implemented. Figure 2(a) shows the user interface utilized throughout this study, providing site-specific (left/right, cheek/forehead/forearm/hand /scalp, site number) and PDT-specific (pre-aminolaevulinic acid (ALA), incubated, and postPDT) image tags.

Images are saved within the specified folder and are accessible via the in-phone preview or through cloud access (iCloud upload). Each folder can be downloaded from the cloud to externally develop image processing algorithms. These image processing algorithms could be directly implemented within the smartphone-based device using the image buffers accessed by the custom iOS application. For this study, the in-phone reporting of FL imaging quantification was used for preliminary evaluation of the acquired images. The images and FL values reported for the phantom, murine, and clinical patient measurements were processed using MATLAB with statistical analysis performed in Minitab 20.1 (Minitab LLC, Pennsylvania, United States).

#### 2.1.2 3D-printed base

The 3D printed base is used for system integration and measurement standardization by physically blocking room light and fixing the sample-to-imager distance. The base is composed of three components: the phone holder, the optics base, and the electronic bay. The phone holder is designed for the iPhone 11 and provides alignment of the two camera apertures within the optical imaging path. The LED ring is attached to the inside of the optics base using a two-sided adhesive. The electronics bay securely attaches the rest of the system components. The phone holder, optics base, and electronics bay are fastened together using M3 machine screws.

Furthermore, a cushioned ring is attached to the exit aperture (the bottom of the optics base) which allows for coupling to the skin. The measurement base fixes the detector-to-imaging plane distance for consistent light irradiation. It is worth emphasizing that the design of the 3D-printed base alongside the cushioned ring forms an isolated lighting environment that allows the system to overcome measurement repeatability issues and enable its use within the clinical environment where ambient light control is challenging.

The detector-to-imaging plane distance was set at 72 mm, which is approximately the minimum focusing distance achievable by the standard camera in the iPhone 11. The output aperture of the measurement base provided a circular field of view of 48 mm. The addition of the cushioned ring limits this aperture to a ∼35  mm field-of-view.

#### 2.1.3 LED ring and electronic components

The system uses a custom LED ring to provide a uniform light source for PpIX peak excitation and white-light illumination. The ring consists of 405 nm LEDs (qty:10, Vishay VLMU3100) and 3000 K white LEDs (qty: 10, Cree J-Series JE2834AWT), providing light intensities of 4.5 and $2.6\;\mathrm{mW}/{\mathrm{cm}}^{2}$ at the measurement output, respectively. The LEDs are mounted on a 1.6 mm aluminum metal-core PCB (2 oz Cu trace) to provide passive thermal management.

**Fig. 3:**
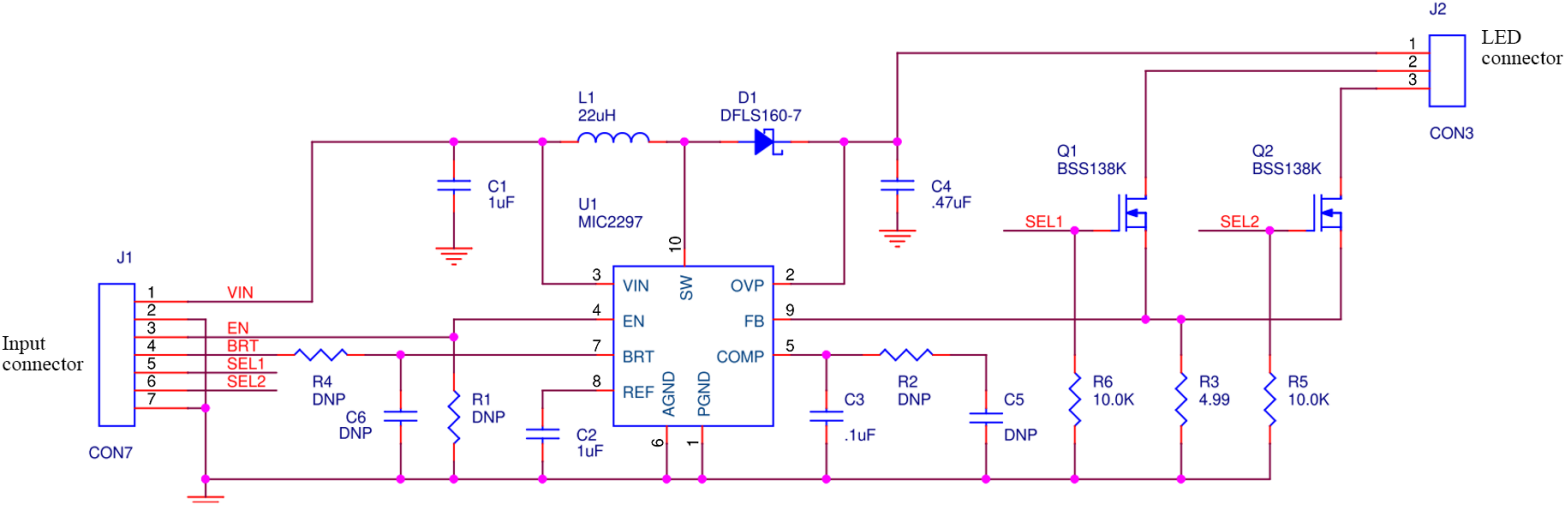
Schematic for the custom current driver board that provides a constant 40 mA current to the LED ring with the ability to enable/disable output and switch between which LED ring is being operated.

The system power electronics consist of a 3.7V lithium-polymer battery (#2011, Adafruit Inc., NYC, New York, United States), USB battery manager (#259, Adafruit Inc.), and custom constant current driver. Fig. 3 shows the schematic of the custom driver, which is based on a MICREL MIC2297 PWM Boost Current Regulator and provides power to the LED ring (40 mA, constant current). The current provided to the LEDs can be adjusted either by changing the R3 resistor value or by varying the voltage at the BRT pin (nominal voltage of 1V) via DC or PWM control. The sDUO fixes the output current to 40 mA using an R3 value of 5 ohms ($\text{current}=V_{\mathrm{ref}}/R3$, where $V_{\mathrm{ref}}=0.2$). The custom current driver adjusts its voltage output to provide the 40 mA constant current to the 405 nm or WL LEDs, which are individually wired in series, requiring a driving voltage of ∼27 and ∼32  V, respectively.

The initiation of the LED driver and switching between the 405 nm and WL illumination is controlled by the Bluetooth-enabled microcontroller (Feather M0 Bluefruit LE, Adafruit Inc.) running Arduino Software. This microcontroller engages in two-way communication with the iOS app to coordinate the activation of the LEDs with their respective imaging modality (FL or WL) as per the process outlined in Fig. 2(b).

The LiPo battery, battery charger, and LED driver board enabled stable light output and rechargeability in a modular package. Implementation of the microcontroller enabled streamlined “single click” acquisition of the FL and WL images to facilitate translation into the clinical environment.

#### 2.1.4 Emission filters and polarizer

The emission filter selection criteria prioritized blocking the 405 nm excitation light, isolating the PpIX fluorescent signal (600 to 700 nm), and minimizing autofluorescence signal from tissue. Both a 600 nm long-pass (600LP RapidEdge, Omega Optical Inc., Brattleboro, Vermont, United States) and 635 nm band-pass (635BP30 RapidBand, Omega Optical Inc.) were used throughout the presented study. Both filters use surface coated dielectrics, providing >90% transmission peaks and ≥OD5 for blocking-band wavelengths. The 600LP filter provides the greatest collection of the PpIX FL signal but is more susceptible to autofluorescence noise from the imaged sample or subject. By contrast, the 635BP filter [635 nm center wavelength, 30 nm full width half maximum (FWHM) bandwidth] provides collection of only the main PpIX FL peak but minimizes the autofluorescence noise. Comparisons of filter performance are found in the results section for phantom, murine, and human patient measurements.

**Fig. 4:**
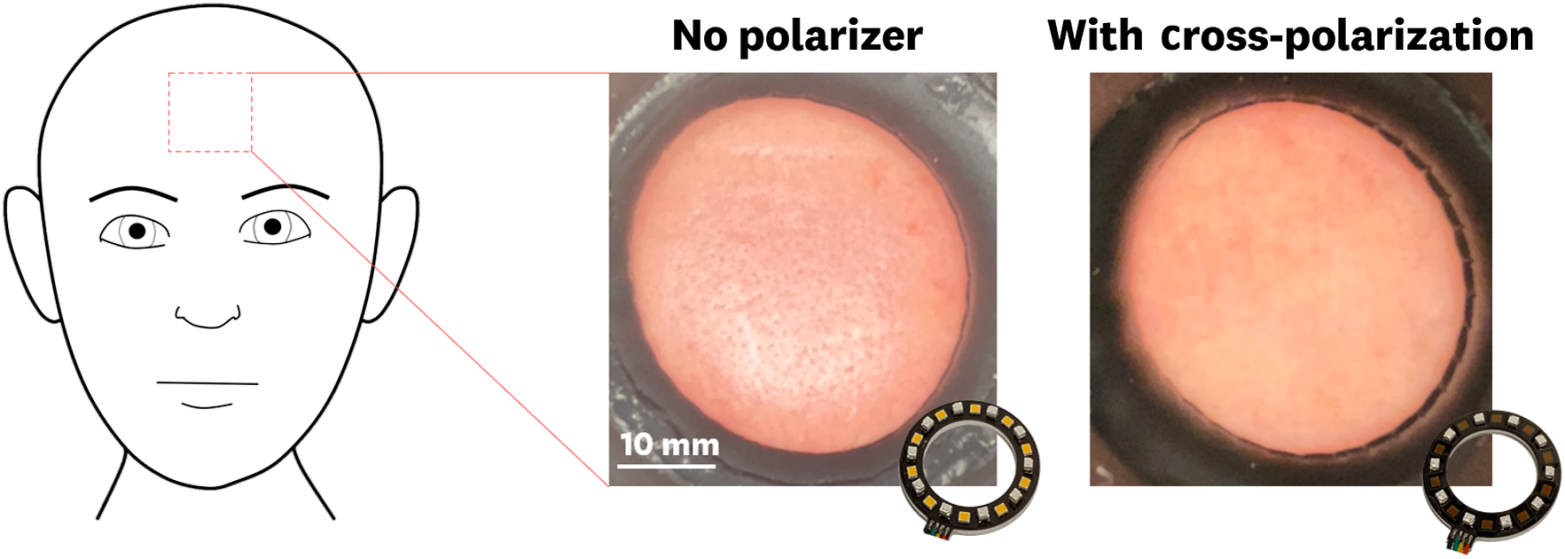
Cross-polarization of the light source and camera eliminates reflections from skin and imaged samples.

A polarizer film was laser cut and placed on a custom 3D-printed mount to provide polarized output for the white LEDs. A corresponding cross-polarized film was placed at the aperture of the ultra-wide camera to minimize skin reflections generated by the LED illumination. An example of how polarization eliminates white-light image reflections from patient skin images is shown in Fig. 4.

### 2.2 System Characterization and PpIX Fluorescence Calibration

To characterize the system’s RAW imaging performance, photon transfer methods^16^ were used for measuring the read-out noise, flat-field, zero offset, dynamic range, and relative pixel quantum efficiency of the standard imaging sensor.

A complete acquisition of the photon transfer curve was performed by imaging a uniform emitting source (A2 LED Tracing Board, Voilamart Inc.) with varying exposure times to measure both the noise-floor and saturation regimes of the standard camera. The RAW images were acquired at a fixed ISO of 400. A centered ROI of 100×100  pixels was used for the average signal and associated variance for each of the images. The photon transfer curve was visualized using a log-log plot of the average signal versus variance. The electron conversion factor was calculated within the shot-noise regime for when the curve slope = 0.5.

Relative quantum efficiency curves for the red, green, and blue imaging pixels were measured using a calibrated monochromator utilizing a tungsten halogen light source (DMC1-07, Optometrics Corp, Massachusetts, United States). The wavelength was scanned from 400-800 nm using a 10 nm step size. A custom 3D-printed holder centered the standard camera aperture to the output of the monochromator. A RAW image was acquired for each wavelength output. A centered ROI of 100×100  pixel for each red, green, and red pixels was used to generate the relative quantum efficiency curves.

PpIX FL calibration measurements involved simulating fluorophore emission by providing a uniform imaging surface with varying radiance using a spectrum that emulated the PpIX emission. This simulated PpIX emission was produced using the 635 nm bandpass filter to image a uniform scattering slab illuminated by white LEDs at varying driving currents. The uniform scattering slab was 3D printed with masked-stereolithography techniques^20^ using ${\mathrm{TiO}}_{2}$ and nigrosine as scatterers and absorbers, respectively ($\mu_{a}=0.06\;{\mathrm{mm}}^{-1}$ and $\mu_{s}^{\prime }=0.8\;{\mathrm{mm}}^{-1}$ @ 630 nm). The varying emission intensities were measured within the optical geometry defined by the dual imager’s optical base, so the calibrated values are relevant to the assembled imager’s geometry. A calibrated photodiode (S120VC, Thorlabs Inc, New Jersey, United States) measured the radiant flux of the simulated FL at the output of the 635BP filter. This measured radiant flux varied in the 0.005 to $2.5\;\mu W/{\mathrm{cm}}^{2}$ range. Subsequently, the iPhone 11 was placed within the same optical geometry and imaged the varying emission intensities. The average pixel values (DN) and the calibrated irradiation measurements were used to extrapolate the average pixel values to an absolute radiometric measurement. The images were acquired at 0.1 s exposure and 400 ISO to extrapolate the FL phantom, murine measurements, and human measurements to estimated absolute radiometric units.

### 2.3 Fluorescence Phantoms

To assess the PpIX concentration sensitivity of the dual imager, a series of liquid FL phantoms containing 1% intralipid, varying amounts of blood (0%, 0.1%, and 1%), and varying amount of PpIX (0.02 to 1000 nM) were prepared. The preparation of the phantoms borrows from methods described by Morois et al.^21^ The negative control solution was 1% intralipid with no PpIX. A bulk quantity of the control solution was prepared by diluting intralipid 20% w/v (NDC 0338-0519-09, Frensius Kabi/Baxter Healthcare) into 1X phosphate buffered saline to achieve 1% w/v. A small quantity of surfactant (Tween 20, P9416-50 mL, Sigma-Aldrich Inc.) was then added at 0.1% v/v to improve the photostability of PpIX in solution. Next, powdered PpIX (P8293-1G, Sigma-Aldrich Inc.) was serially diluted 3:1 in dimethyl sulfoxide using a starting concentration of 1200  μM. A fixed volume aliquot from each serial dilution was then pipetted into $25\;{\mathrm{cm}}^{2}$ rectangular cell culture flasks (COR-430168, Corning) previously filled with ∼50  mL of the 1% intralipid background solution. Each flask was then rocked to mix the PpIX, filled completely with the 1% intralipid solution to fill the container volume, and sealed with the flask cap. Transfer from Eppendorf tubes and dilution into the cell culture flasks constituted a fixed dilution factor of 400. Final PpIX concentrations of the liquid phantoms were 1000, 333, 111, 37, 12, 4.1, 1.4, 0.46, 0.15, 0.051, 0.017, and 0 nM.

Images of the varying concentrations were acquired in triplicate for both the 600LP and 635BP filter configurations of the sDUO. The room lights were turned off for the measurements to reduce the scattered light in the liquid phantoms. After completion of the 1% intralipid solution measurements, bovine blood was added to each flask to provide a 0.1% blood baseline. The flasks were manually agitated to achieve homogeneous distribution of the blood. The 1% intralipid + 0.1% blood flasks were imaged before adding more bovine blood to achieve a 1% blood solution. These 1% intralipid + 1% blood solutions were manually agitated and imaged.

The acquired images were analyzed by taking a centered 100×100  pixels ROI for each image with the reported values generated from the average of the triplicates. The reported FL image quality metric, contrast-to-variability ratio (CVR), is defined as $\mathrm{CVR}=(I_{\mathrm{PpIX}}^{\mathrm{FL}}-I_{\text{Control}}^{\mathrm{FL}})/\sqrt{\sigma_{\mathrm{PpIX}}^{2}+\sigma_{\text{Control}}^{2}}$, where $I_{\mathrm{PpIX}}^{\mathrm{FL}}$ and $I_{\text{Control}}^{\mathrm{FL}}$ are the averaged FL intensities for the PpIX liquid phantoms and the control, alongside their respective variances $\sigma_{\mathrm{PpIX}}^{2}$ and $\sigma_{\text{Control}}^{2}$, respectively.^22^ We define the lower limit of detection for the liquid phantoms for measurements resulting in a CVR>1.5. The CVR metric is preferred over standard signal-to-noise calculations as it provides a better estimation of FL contrast performance by accounting for the signal and variance of both the FL and control regions in the metric calculation.^23^^,^^24^ The linear range of each phantom was determined as the concentration range that provides an $R^{2}>0.95$ for a power fit of the data.

### 2.4 Pre-clinical In-vivo Murine Measurements

All animal procedures were carried out following a protocol approved by the Dartmouth College Institutional Animal Care and Use Committee. The nude mouse model was used to examine the ability of the system to capture *in-vivo* production of PpIX. The measurements were performed on three athymic nude mice fed a low-chlorophyll diet to minimize tissue autofluorescence. For imaging, the mice were placed under anesthesia and onto a heated pad to regulate their body temperature; the temperature was monitored using an Infra-red (IR) camera (TG165, FLIR Systems Inc.) and kept in the 32°C to 36°C range. AMELUZ^®^ (Biofontera Inc., Boston, MA), a 10% ALA hydrochloride gel, was topically applied to the backs of the mice to contain an ALA-applied area and a control area within the imaging field-of-view. Images of the mice were taken at baseline (before the application of ALA) and at 10, 30, 60, and 180 min. A 100×100  pixel ROI was used for the ALA-applied area and a control area for quantifying the accumulation of PpIX at the measured timepoints. An additional 3D-printed bracket was used to standardize the imaging distance and fix the angle of the sDUO for the mice measurements.

### 2.5 Clinical Measurements of PpIX-based PDT

All human imaging was performed on patients who were recruited from the standard patient population appearing with AKs at the Dartmouth-Hitchcock Department of Dermatology Clinic and gave informed consent before enrollment. The study was approved by the Dartmouth-Hitchcock Health Institutional Review Board and registered with ClinicalTrials.gov (NCT03805737). Cleaning of the treatment area (face, forehead, and scalp) using a 70% isopropyl solution was followed by topical application of a 10% ALA gel (Ameluz^®^, Biofrontera). Following ALA application, patients were incubated under room-light conditions for 30 mins, with the area of treatment uncovered. Following the ALA incubation, light irradiation was performed on the treatment areas. For the study, patients either received 10 min of high irradiance red-lamp treatment or 2 hrs of indoor daylight treatment. Irradiance spectra and PpIX-equivalent dose are provided in the Supplementary Material following the methods described in Ruiz et al.^25^ In brief, the effective PpIX dose calculations show that the indoor-daylight treatment provides a higher dose (2×) for depths below <250  μm,^26^ which corresponds to the location of the upper blood dermis in a seven-layer skin model.^27^ The delivered PpIX-effective dose is equivalent within 20% in the 350 to 1000  μm range, with an equal dose delivered at 700  μm between the two treatment regimens.

The use of the sDUO in this study was aimed at understanding (1) the heterogeneity of PpIX accumulation between patients and between sites, (2) the mechanistic differences between the red-lamp and indoor-daylight treatments with emphasis in differences in the photobleaching rates, and (3) the measured incubated and post-PDT FL to AK lesion clearance outcomes. Details of the clinical design, patient population, clinical outcomes, and efficacy equivalency between indoor-daylight and red-lamp PDT are published in Ruiz et al.^25^ Briefly, the primary outcome was therapeutic efficacy (AK lesion clearance rate), defined as the reduction in the number of AK lesions between the baseline (day of treatment) and the follow-up visit (1-month and 6-month post-treatment). The AK lesions were mapped and counted by physicians for the treatment region (face, forehead, and scalp) for both the baseline and 1-month, and 6-month follow-ups. The clinical measurements presented in this paper focus on the data from 15 patients that were imaged using both configurations of the sDUO (600LP and 635BP). For these patients, triplicate measurements were taken for each treatment site (cheek, forehead, and scalp) at the three stages of treatment (pre-ALA application, at 30-min incubation, and post-PDT irradiation), so nine FL and nine white-light images were generated per treatment site. The acquired images were centered at each treatment site rather than at each identified AK lesion. We used these “site-centered” FL measurements, rather than lesion-specific measurements, due to the practical limitations of tracking the individual AK lesions. Furthermore, PDT treatment of AKs is generally performed for a whole site and not localized to individual lesions, enabling the use of these site-centered measurement of PpIX accumulation in the standard clinical workflow.

**Fig. 5:**
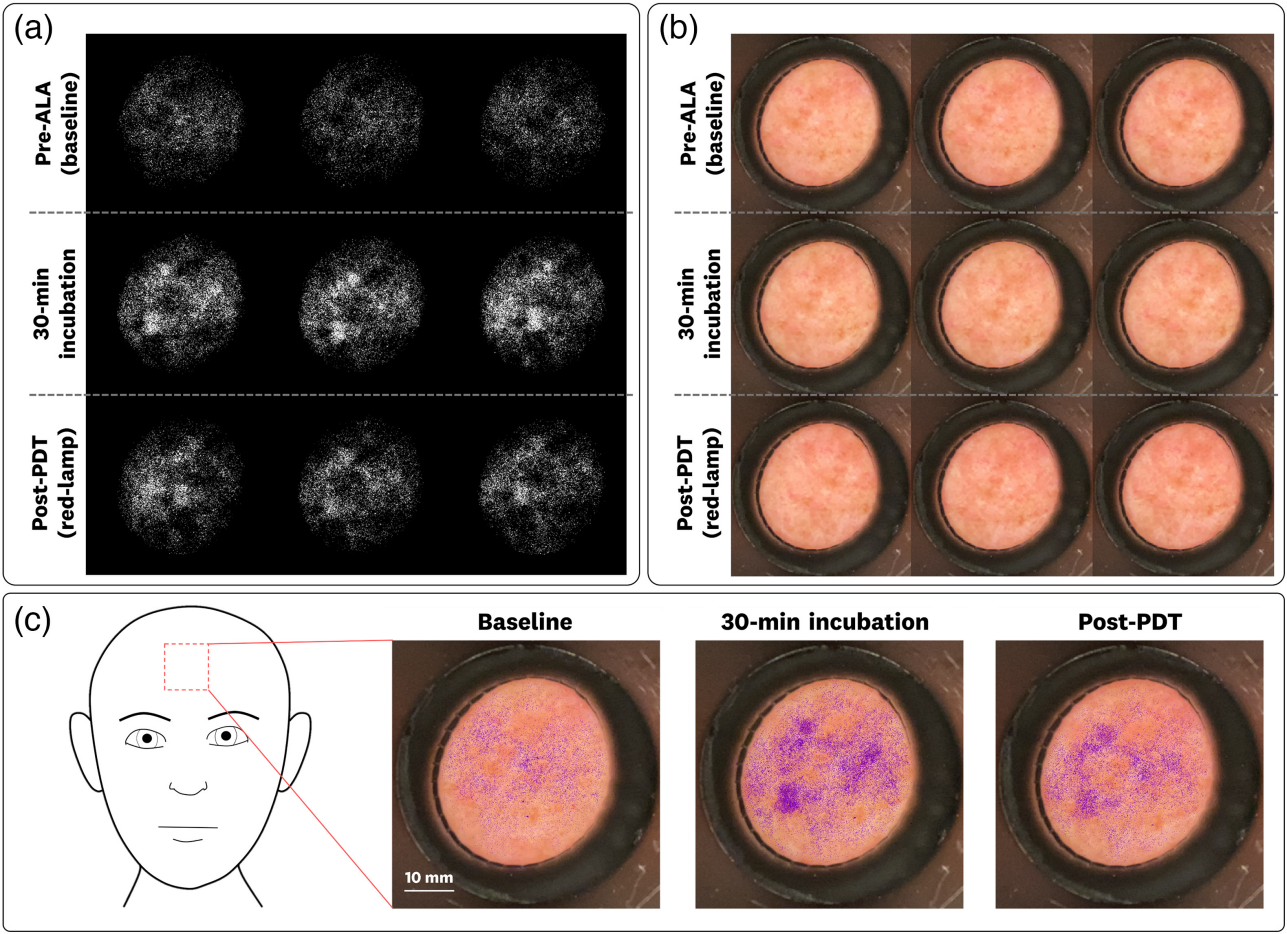
Example of a complete data set for an imaged patient site (forehead measurement is shown) in which each PDT treatment timepoint is imaged three times. (a) FL images show an increase in FL for the 30-min incubation and subsequent photobleaching following the 10-min red-lamp irradiation. (b) The associated white-light images corresponding to the FL images in panel (a). (c) FL overlay images using a purple hue for visual clarity. All FL images and overlays use the same intensity mapping, allowing for cross-image comparison.

These patient images were analyzed by taking a circular ROI (250 pixel diameter, ∼10  mm) centered on the imaged aperture. The displayed FL images used a threshold of baseline (0) as the minimum mean calculated for each site and an upper limit (1) of 106% of the maximum calculated mean of each treatment site. An example patient image set is shown in Fig. 5 for the PDT treatment of a patient forehead.

The FL images [Fig. 5(a)] have corresponding white-light images [Fig. 5(b)], which can be co-registered to provide an FL overlay [Fig. 5(c)]. For this study, we use a purple hue for the FL overlay mapping to provide visual clarity. The example dataset (Fig. 5) shows the FL accumulation and subsequent photobleaching from the 30-min incubation period and 10 min red-lamp irradiation, respectively. To determine the statistical significance in the calculated FL values, a paired t-test was used on the average values for the calculated ROIs. Statistical significance was considered at a p-value of less than 0.05. Linear regression fits were used to study the correlation of the measured FL to the site-specific AK lesion clearance rates. The quality metric of contrast-to-standard error ratio (CSER) was used for comparing the performance of the 600LP and 635BP filters over the measured sites. The CSER allows for a quality metric that accounts for the averaging over the sites, where $\mathrm{CSER}=(I_{\text{Incubated}}^{\mathrm{AVG}}-I_{\text{baseline}}^{\mathrm{AVG}})/\sqrt{{\mathrm{SE}}_{\text{Incubated}}^{2}+{\mathrm{SE}}_{\text{baseline}}^{2}}$. Reported absolute irradiance values ($\mathrm{nW}/{\mathrm{cm}}^{2}$) are extrapolated from the PpIX FL calibration with a power fit of $I_{\mathrm{rad}}=0.419*{\mathrm{DN}}^{0.9752}$, where $I_{\mathrm{rad}}$ is the radiometric irradiance in $\mathrm{nW}/{\mathrm{cm}}^{2}$ and DN is the baselined pixel intensity using the 0.1 s exposure, 400 ISO image acquisition settings. This power fit had a goodness of fit $R^{2}=0.999$ for DN values >10. The estimated changes in PpIX concentrations are extrapolated from the liquid phantom measurements (1% intralipid + 1% blood) with a power fit of ${\mathrm{PpIX}}_{nM}=0.1138*{\mathrm{DN}}^{1.555}$, where ${\mathrm{PpIX}}_{nM}$ is the estimated concentration of PpIX (in nM) and DN is the baselined pixel intensity using the 0.1 s exposure, 400 ISO image acquisition settings. The power fit had a goodness of fit $R^{2}=0.993$.

## 3 Results

### 3.1 System Characterization and PpIX Fluorescence Calibration

**Fig. 6:**
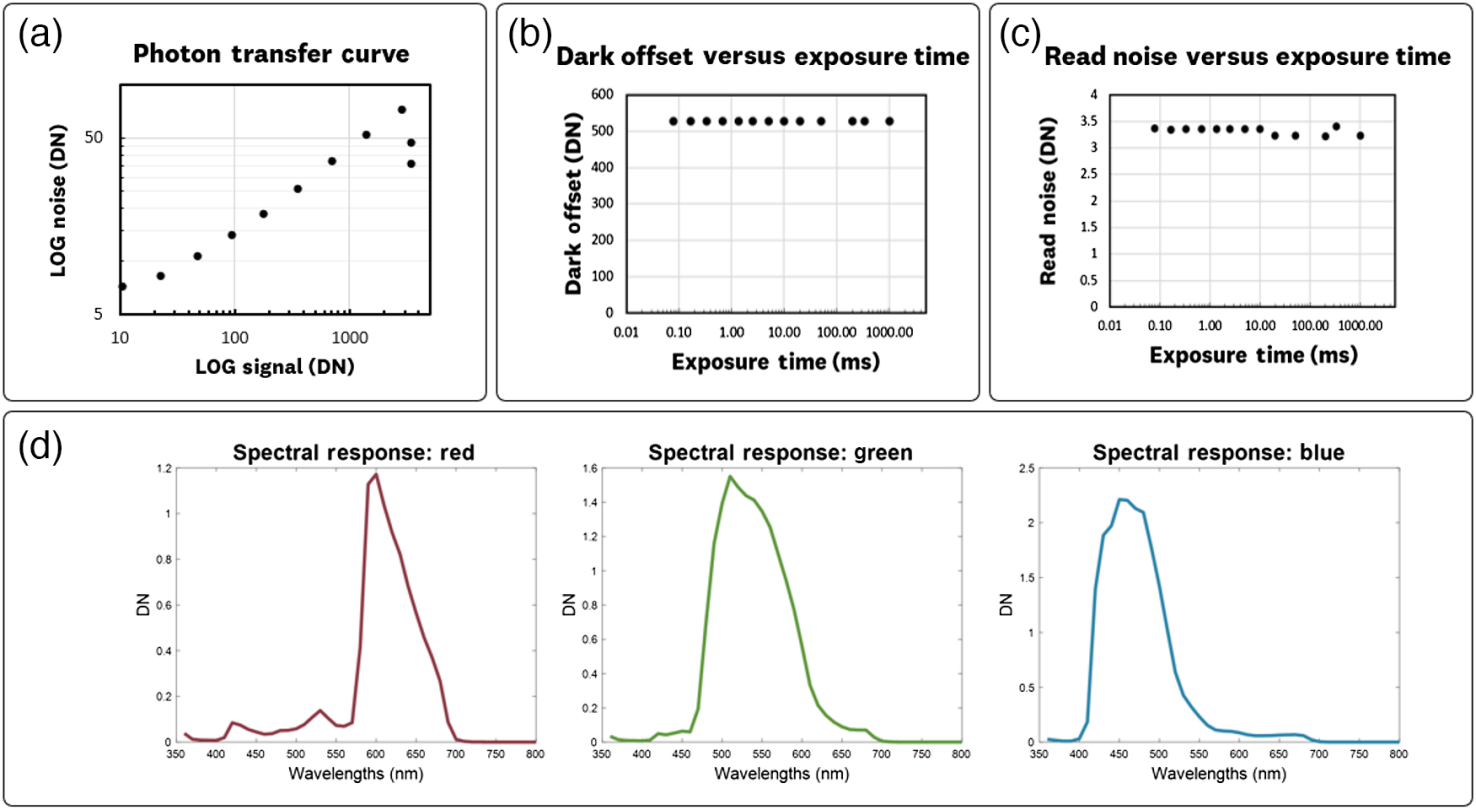
Photon transfer measurement results for the iPhone 11’s standard camera. (a) The photon transfer curve shows the expected read noise, shot noise, fixed-pattern noise, and full-well regimes. (b) The dark offset versus exposure time plot shows a consistent value of ∼527 counts (DN). (c) Read-noise versus exposure time plot shows a 3.4 DN read-noise. (d) Relative spectral response plots for the red, green, and blue pixels.

The standard camera of the iPhone 11 was characterized using photon transfer methods to determine its ability to perform quantitative imaging. Figure 6 shows the plotted results for the photon transfer curve, dark offset, read noise, and spectral quantum efficiency measurements.

The photon transfer curve [Fig. 6(a)] shows the expected read-noise, shot noise, fixed-pattern noise, and full well regimes.^16^ A dark-offset of 527 DN and a full-well capacity of 4095 DN are measured. From the shot-noise limited regime, we obtain an electron conversion factor of $∼0.52\;{\mathrm{DN}/e}^{-}$, indicating that each digital number above the dark-offset corresponds to about two electrons read out by the electric circuit. The photon transfer curve also indicated that the system should have a linear pixel response to an increased photon flux.

The dark offset versus exposure time plot [Fig. 6(b)] confirms a constant 527 DN dark offset value for varying exposure times. This result, a constant dark offset with changing exposure, indicates that the main sensor has a built-in dark current correction.

The read-noise vs. exposure time plot [Fig. 6(c)] shows a constant read-out noise level of ∼3.4  DN. Given this constant read-out noise, determined full well capacity of 4095 DN, and dark offset of 527 DN, the dynamic range of the iPhone 11 standard sensor is estimated as (4095 to 527)/3.4 = 1049.

The spectral response plots for the red, green, and blue imaging pixels [Fig. 6(c)] show typical band-pass peaks for RGB bayer-filter cameras.^18^ These results indicate that for PpIX FL signal (600 to 700 nm range) the red-pixels provide the best signal acquisition while limiting the excitation and tissue autofluorescence noise.

**Fig. 7:**
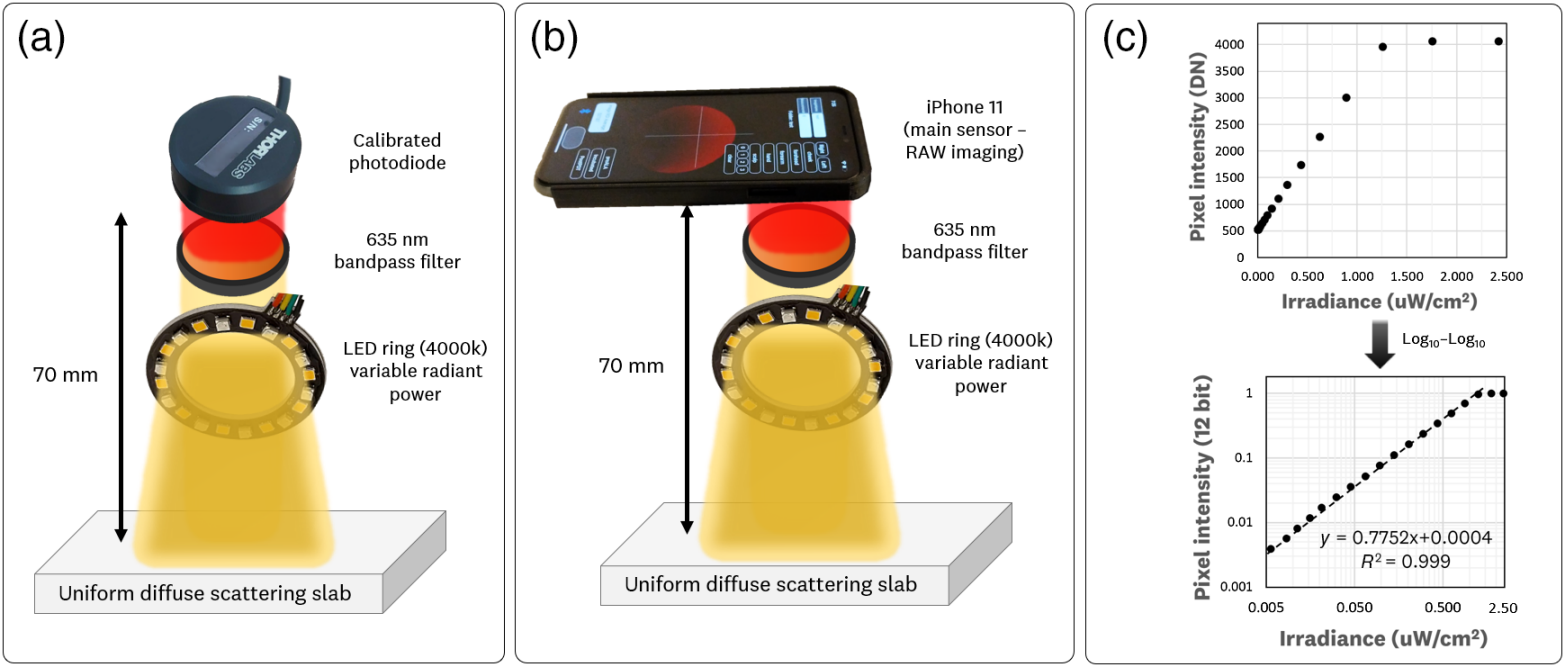
PpIX FL calibration measurements for RAW imaging quantification. The PpIX FL calibration involves simulating fluorophore emission by providing a uniform imaging surface with varying radiance using the 635 nm bandpass filter to image a uniform scattering slab illuminated by white LEDs at varying driving currents. The varying irradiances were measured within the optical geometry of the sDUO optical base by (a) the calibrated photodiode to allow for radiant flux measurement of the simulated FL and (b) the iPhone 11 main camera sensor. (c) The resulting pixel intensity (DN) versus irradiance ($\mu W/{\mathrm{cm}}^{2}$) calibration curves and respective (d) log10-log10 plots are shown alongside the linear fit (y=0.7752x+0.0004) with $R^{2}=0.999$.

The results from the simulated radiometric PpIX FL calibration are shown in Fig. 7. The calibrated photodiode [Fig. 7(a)] measured the radiant exitance that is emitted/scattered by the diffuse slab and filtered through the 635BP filter. These radiometric values (calibrated for the 635 nm band-pass peak) were used alongside the average pixel values from the iPhone 11 standard camera [Fig. 7(b)] to generate the pixel intensity vs. irradiance plot [Fig. 7(c)].

This plot shows red-pixel sensor saturation for irradiances $>1.25\;\mu W/{\mathrm{cm}}^{2}$. The $\log_{10}\text{-}\log_{10}$ plot shows that the camera response linearity extends to the 0.005 to $1.25\;\mu W/{\mathrm{cm}}^{2}$ range, with a linear fit equation given by y=0.7752x+0.0004 and goodness of fit metric $R^{2}=0.999$. This pixel intensity versus irradiance radiometric calibration curve was acquired at 0.1 s exposure and 400 ISO, such that these results can be used to extend the FL emission of the phantom, murine, and human measurements into radiometric units. The photon transfer characterization and radiometric irradiance calibration show the capability to provide measured ROI units with ±0.1  DN and $\pm 0.1\;\mathrm{nW}/{\mathrm{cm}}^{2}$ precision, respectively.

### 3.2 Fluorescence Phantoms

**Fig. 8:**
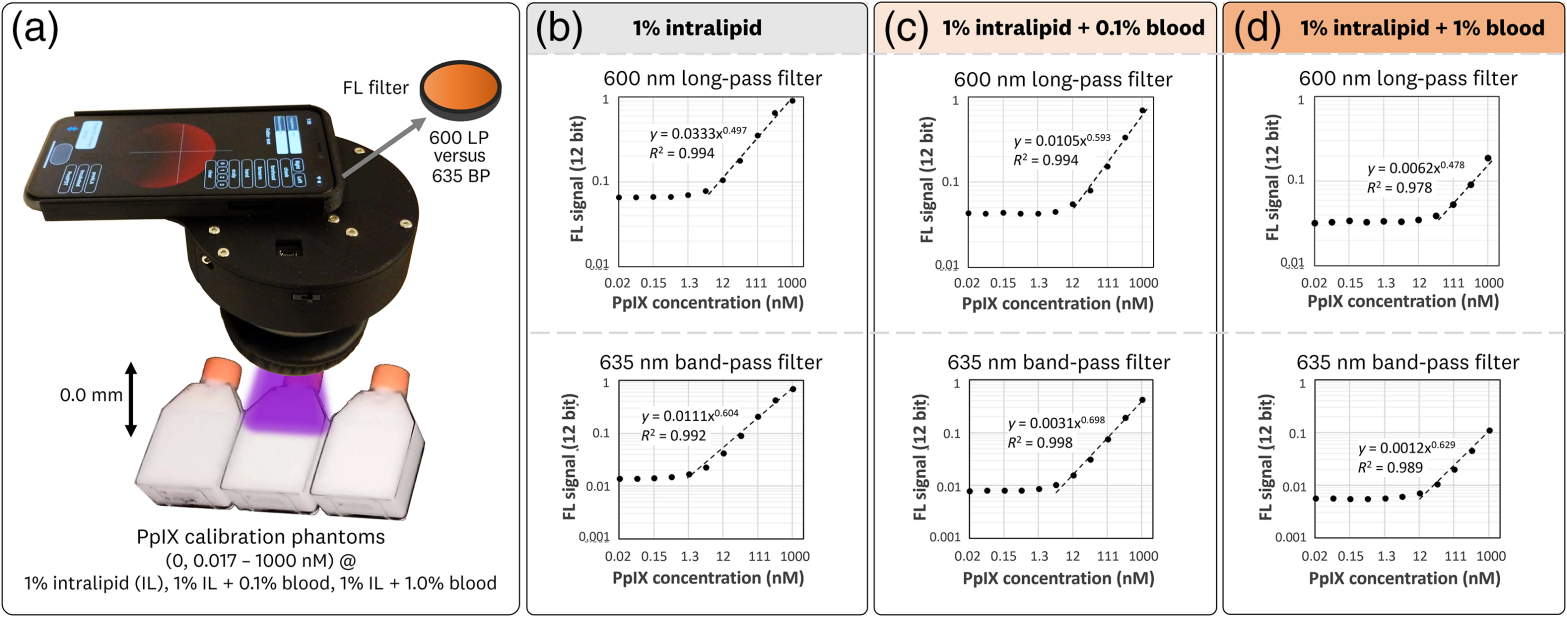
PpIX FL phantom measurements to assess the PpIX concentration sensitivity of the sDUO. (a) Series of liquid FL phantoms containing 1% intralipid, varying amounts of blood (0%, 0.1%, and 1%), and varying amounts of PpIX (0.02 to 1000 nM) were imaged with both the 600LP and 635BP filter configurations. The plots generated from the average FL signal for the ROI of the acquired images are shown for the (b) 1% intralipid, (c) 1% intralipid + 0.1% blood, and (d) 1% intralipid + 1% blood measurements. The error bars are smaller than the size of the plotted points, so they are omitted for visual clarity.

The results from the preparation and imaging of the liquid FL phantoms are shown in Fig. 8. The varying PpIX concentrations (0.017 to 1000 nM, and control) were imaged with both the 600LP and 635BP filter configurations [Fig. 8(a)]. The average ROI results are plotted for the 1% intralipid [Fig. 8(b)], 1% intralipid + 0.1% blood [Fig. 8(c)], and 1% intralipid + 1% blood [Fig. 8(d)] solutions. These log-log plots of the measured FL signal versus PpIX concentration facilitate the visualization of the linearity range for each solution. As a broad trend, these measurements show that the increase in blood content decreased the sensitivity of the measurement, such that the linear range becomes smaller.

The 635BP filter configuration measured a lower limit of detection at 0.45, 1.4, and 4.1 nM for the 1% intralipid, 1% intralipid + 0.1% blood, and 1% intralipid + 1% blood solutions, respectively. The corresponding linearity range was measured as 1.4 to 1000 nM, 4.1 to 1000 nM, and 12−1000  nM, respectively. Power fits and $R^{2}$ values for these linear ranges were $y=0.011x^{0.604}$ ($R^{2}=0.99$), $y=0.0031x^{0.698}$ ($R^{2}=0.99$), and $0.0012x^{0.629}$ ($R^{2}=0.99$), respectively.

The 600LP filter configuration measured a lower limit of detection at 1.4, 4.1, and 37 nM for the 1% intralipid, 1% intralipid + 0.1% blood, and 1% intralipid + 1% blood solutions, respectively. The corresponding linearity range was measured as 4.1 to 1000 nM, 12 to 1000 nM and 37 to 1000 nM. Power fits and $R^{2}$ values for these linear ranges were $y=0.035x^{0.479}$ ($R^{2}=0.99$), $y=0.0105x^{0.592}$ ($R^{2}=0.99$), and $y=0.0062x^{0.478}$ ($R^{2}=0.98$).

For these results, the 635BP filter configuration provided ∼3× greater sensitivity (lower limit of detection) compared with the 600LP configuration for the 1% intralipid and 0.1% blood solutions. For the 1% intralipid + 1% blood solution, the 635BP filter configuration achieved a ∼9× greater sensitivity. This indicates that the 635BP filter provides a better signal to variability performance by eliminating background autofluorescence while preserving the collection of PpIX signal. As a comparison, the CVR for the 37 nM solution for the 635BP and 600LP configurations were ${\mathrm{CVR}}_{635\mathrm{BP}}=[232,48.0,15.4]$ and ${\mathrm{CVR}}_{600\mathrm{LP}}=[39.1,23.1,2.7]$ for the 1% intralipid, 1% intralipid + 0.1% blood, and 1% intralipid + 1% blood solutions, respectively.

### 3.3 *In-vivo* Murine Measurements

**Fig. 9:**
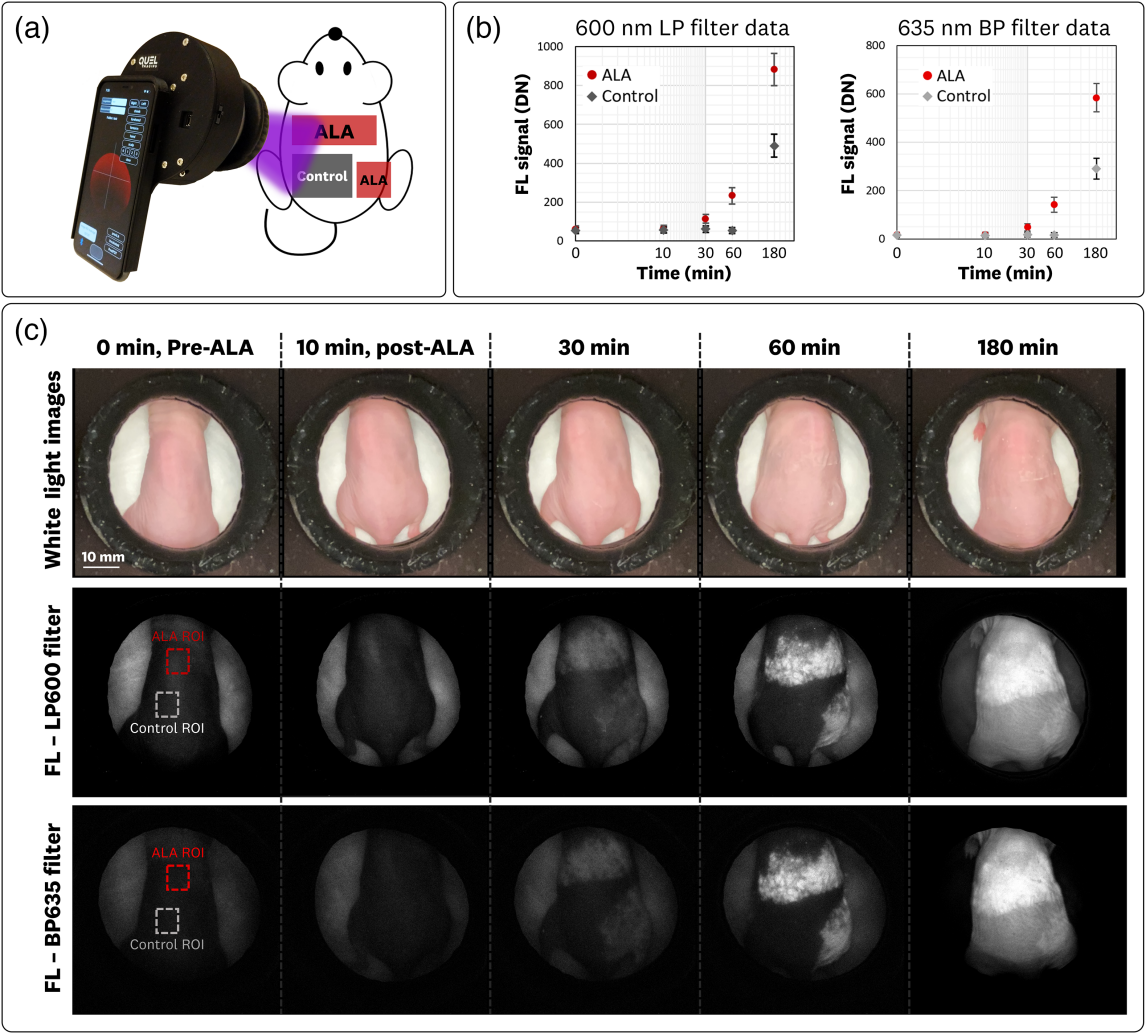
*In-vivo* murine sDUO measurements using the 600LP and 635BP filter configurations. (a) Testing set-up for *in-vivo* imaging of PpIX accumulation in athymic nude mice. (b) Pixel intensity versus time plot for the ALA and control ROI shows the monotonic increase in FL due to the PpIX accumulation in the ALA region and a relatively flat response in the control region; a moderate increase in FL is observed in the control region at 180 min of incubation as the mice begin systemic accumulation of PpIX. Error bars report the standard deviation from the average across the mice measurements. (c) Resulting WL and FL images for both 600LP and 635BP filter configurations.

The results from the athymic nude mice measurements are shown in Fig. 9. The sDUO was used with both the 600LP and 635BP filter configurations to image the PpIX accumulation from the topical application of ALA [Fig. 9(a)].

The averaged pixel ROI values for the mice measurements (n=3) are plotted in Fig. 9(b) from the corresponding ALA and control ROIs shown in Fig. 9(c). The plots show the monotonic increase in FL for the ALA-applied region with no increase in the control signal for the first 60 min of incubation. A moderate increase in FL is observed in the control region at 180 min of incubation as the mice begin the systemic accumulation of PpIX. The accumulation and systemic generation of PpIX was also observed for the mice imaged with the first-generation imager.^15^

The associated WL and FL images for both the 600LP and 635BP filter configurations are shown in Fig. 9(c). The FL images use the same window/level to allow for cross-comparison of the images. The visual accumulation at the 30 min timepoint is clearly visualized from these measurements. The systemic accumulation at the 180 min timepoint is also clearly visualized. The 635BP FL images measured a lower background signal along with lower PpIX FL signal.

The measured CVR metrics for the 600LP and 635BP filter configurations at the [10, 30, 60, and 180 min] timepoints were ${\mathrm{CVR}}_{600\mathrm{LP}}=[0.31,2.6,5.5,5.4]$ and ${\mathrm{CVR}}_{635\mathrm{BP}}=[0.19,2.5,5.4,5.7]$. These measurements show almost identical contrast-to-variance performances from both filter configurations. This result differs from the liquid phantom measurements, in which the 635BP filter outperformed the 600LP CVR performance by >2×. This highlights the need to characterize the filter performance for the intended application. For the murine measurements, the relatively low autofluorescence background and localized PpIX accumulation resulted in similar performances from the two filter configurations.

### 3.4 Clinical Measurements of PpIX-based PDT

A total of 15 patients undergoing treatment of AKs were imaged with the sDUO using both the 600LP and 635BP filter configurations. Six of these patients received standard red-lamp PDT and seven received indoor-daylight PDT. A total number of 33 lesion sites were imaged (14 cheeks, 9 foreheads, and 10 scalps).

The average baseline and incubated FL signal emission for the 33 lesions, using the 600LP filter configuration, was measured as 191.7±33.7  DN ($70.5\pm 12.1\;\mathrm{nW}/{\mathrm{cm}}^{2}$) and 195.3±34.8  DN ($71.8\pm 12.5\;\mathrm{nW}/{\mathrm{cm}}^{2}$), with a paired t-test P-value of 0.07. For the 635 BP filter configuration, the measurements were 54.9±9.1  DN ($20.8\pm 3.4\;\mathrm{nW}/{\mathrm{cm}}^{2}$) and 60.1±10.5  DN ($22.8\pm 3.9\;\mathrm{nW}/{\mathrm{cm}}^{2}$), with a paired t-test P-value of <0.001. These results indicate that sDUO was able to detect the increase in FL signal from PpIX accumulation during the 30-min incubation period. For these paired baseline-incubation measurements, the 635BP filter detected similar changes in the average FL while providing lower variability in measurements when compared with the 600LP filter. Furthermore, the measured CSER for the 600LP and 635BP filter configurations for the baseline and 30 min incubated timepoints were ${\mathrm{CSER}}_{600\mathrm{LP}}=\frac{195.3-191.7}{\sqrt{6.1^{2}+5.9^{2}}}=0.43$ and ${\mathrm{CSER}}_{635\mathrm{BP}}=\frac{60.1-54.9}{\sqrt{1.9^{2}+1.6^{2}}}=2.1$. These values, calculated from the average of the 33 imaged lesions, further suggest that the enhanced performance from the 635BP filter is related to a lower variance in the measurements.

For the lesions treated with red-lamp PDT, the incubated and post-PDT FL signal emission was measured, for the 600LP filter configuration, as 197.5±43.1  DN ($72.5\pm 15.4\;\mathrm{nW}/{\mathrm{cm}}^{2}$) and 188.2±42.0  DN ($69.3\pm 15.1\;\mathrm{nW}/{\mathrm{cm}}^{2}$) with a paired t-test P-value of 0.01. For the 635BP filter configuration, the measurements were 61.1±12.2  DN ($23.1\pm 4.5\;\mathrm{nW}/{\mathrm{cm}}^{2}$) and 58.4±12.4  DN ($22.1\pm 4.6\;\mathrm{nW}/{\mathrm{cm}}^{2}$) with a paired t-test P-value of 0.03. These results indicate that the sDUO was able to detect the photobleaching associated with the 10-min red-lamp irradiation. For these measurements, the comparable performance from the filter configurations might be attributed to relatively small changes in background signal related to the 10-min red-lamp irradiation, where the 600LP, despite higher measurement variance, maximizes the FL signal collection

For the lesions treated with indoor-daylight PDT, the incubated and post-PDT FL signal emission was measured, for the 600LP filter configuration, as 192.9±24.1  DN ($70.1\pm 8.6\;\mathrm{nW}/{\mathrm{cm}}^{2}$) and 205.6±34.0  DN ($75.5\pm 12.2\;\mathrm{nW}/{\mathrm{cm}}^{2}$) with a paired t-test P-value of 0.059. For the 635BP filter configuration, the measurements were 58.1±8.1  DN ($22.0\pm 3.0\;\mathrm{nW}/{\mathrm{cm}}^{2}$) and 66.0±15.0  DN ($24.9\pm 5.5\;\mathrm{nW}/{\mathrm{cm}}^{2}$) with a paired t-test P-value of 0.005. These results indicate that the sDUO measured an increase in the PpIX FL signal after the 2 hr irradiation. This implies that the rate of PpIX production surpasses the rate of photobleaching from the indoor-daylight PDT. In contrast to the red-lamp treated lesions, the 600LP underperformed the 635BP filter within the indoor-daylight measurements. The long 2 hr treatment times could allow for larger changes in the background conditions, contributing to a higher variance in the 600LP measurement compared with its PpIX FL collection advantages.

**Fig. 10:**
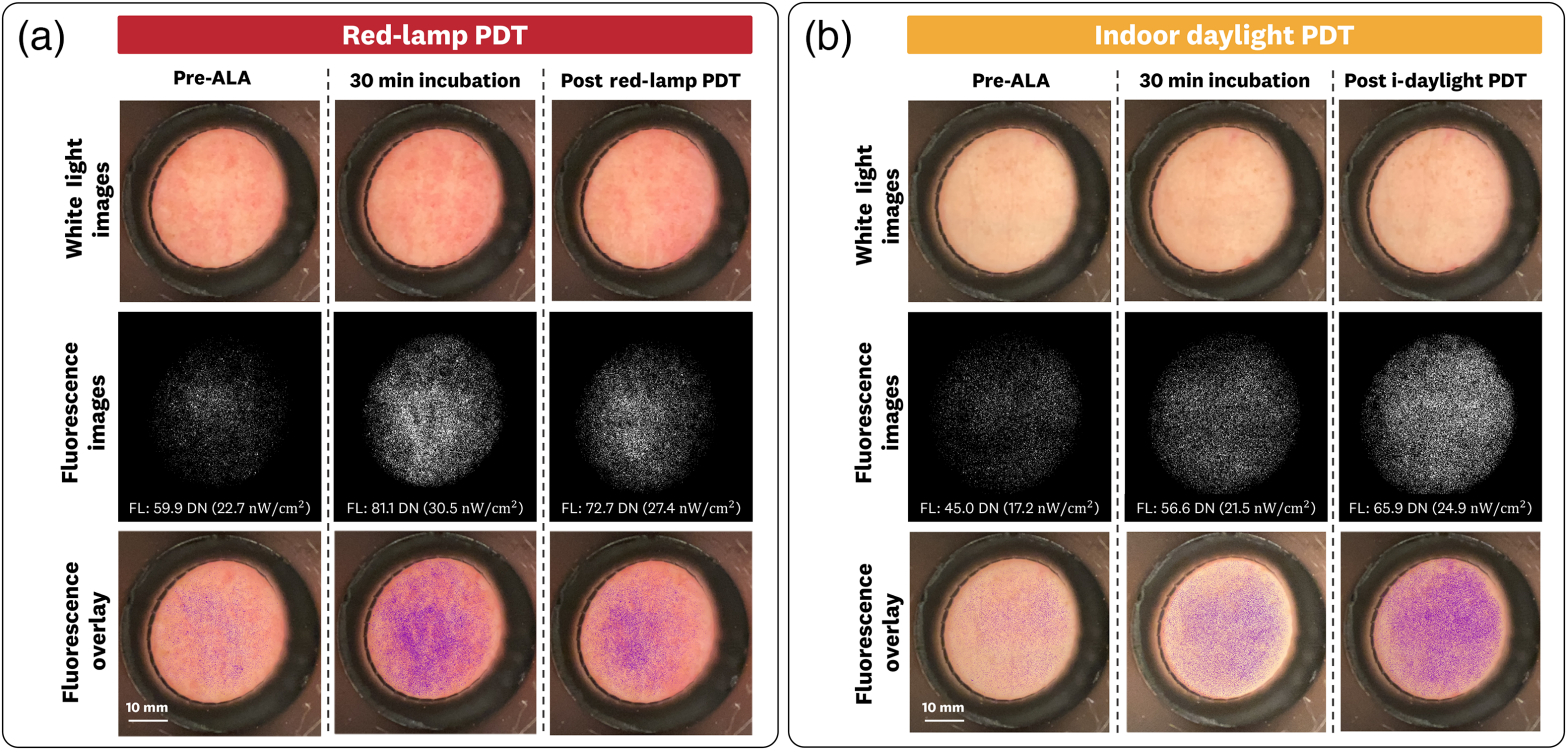
Clinical measurements (WL, FL, and overlay images) of patient foreheads undergoing PpIX-based PDT treatment using red-lamp and indoor-daylight irradiation. (a) The red-lamp treated patient shows an increase in FL signal followed by photobleaching of the signal from the lamp irradiation (b) FL images are sequentially captured during measurement. (b) The daylight-treatment patient shows a continued increase of FL signal, indicating that the rate of PpIX production surpasses the rate of photobleaching from the indoor-daylight PDT. FL images use the 635BP filter configuration with a consistent window/level for each site. FL intensity values reported are the average values over each respective image set from a 200 pixel circular ROI (∼20  mm diameter).

Representative patient images measured using the sDUO (635BP configuration) for red-lamp and indoor-daylight treatment are shown in Fig. 10. The red-lamp PDT images [Fig. 10(a)] show an increase in the FL signal due to PpIX incubation followed by photobleaching from the lamp irradiation. The baseline, incubated, and post-PDT values for the red-PDT treated site were 59.9±2.0  DN ($22.7\pm 0.8\;\mathrm{nW}/{\mathrm{cm}}^{2}$), 81.1±3.6  DN ($30.5\pm 1.5\;\mathrm{nW}/{\mathrm{cm}}^{2}$), and 72.7±1.0  DN ($27.4\pm 0.4\;\mathrm{nW}/{\mathrm{cm}}^{2}$), respectively. Using the 1% intralipid + 1% blood liquid phantom results, we can extrapolate these changes of FL as an estimated PpIX increase of 40 nM and a decrease of 16 nM for the incubation and post-PDT timepoints, respectively.

The indoor-daylight PDT images [Fig. 10(b)] show an increase in FL signal due to the PpIX incubation followed by a continued increase in signal after the 2 hrs of irradiation. The baseline, incubated, and post-PDT values for the indoor-daylight PDT lesion were 45.0±0.6  DN ($17.2\pm 0.3\;\mathrm{nW}/{\mathrm{cm}}^{2}$), 56.6±1.1  DN ($21.5\pm 0.5\;\mathrm{nW}/{\mathrm{cm}}^{2}$), and 65.9±1.2  DN ($24.9\pm 0.5\;\mathrm{nW}/{\mathrm{cm}}^{2}$), respectively. Using the 1% intralipid + 1% blood liquid phantom results, we can extrapolate these changes of FL as an estimated PpIX increase of 18 nM and a continued increase of 16 nM for the incubation and post-PDT timepoints, respectively.

The baseline and 30 min incubated FL intensity values showed no statistical difference between the cheek, forehead, and scalp sites for both filter configurations. The difference in FL between paired baseline and incubated measurements, although not statistically significant for our sample size, hinted at the scalp site having lower incubated values, which might correlate with the lower lesion clearance rates reported for the clinical outcomes of the study.^25^

#### 3.4.1 Linear correlation AK lesion rates vs. imaged fluorescence

**Fig. 11:**
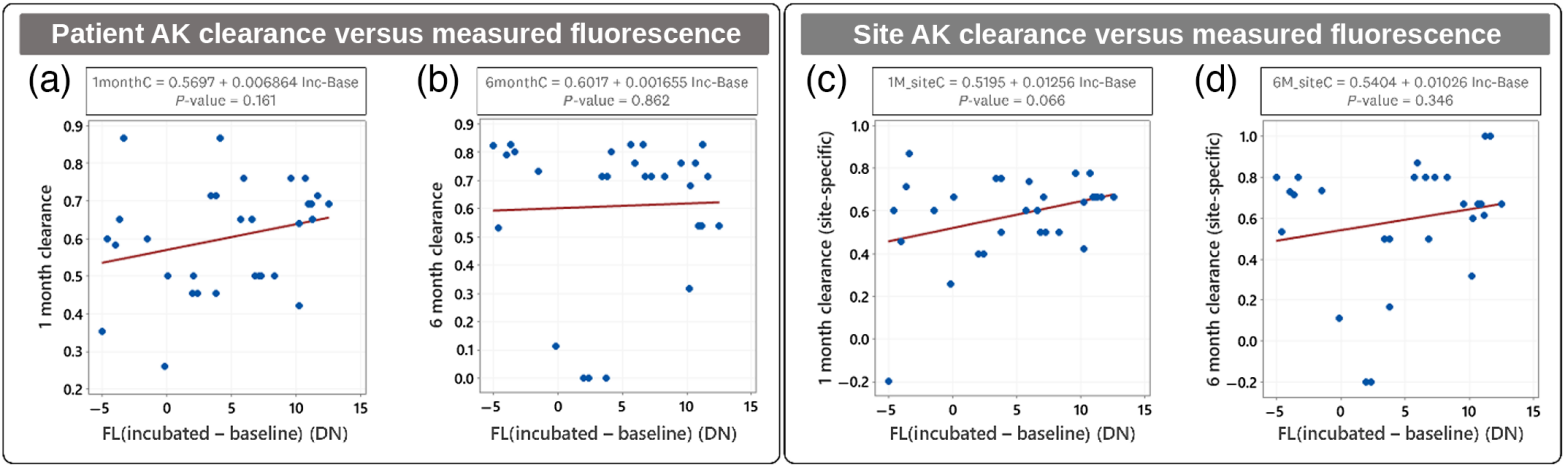
AK lesion clearance correlation to measured FL intensity. (a) 1 month and (b) 6 month patient-specific AK lesion clearance rate data and correlation fits. (c) 1 month and (d) 6 month site-specific (face, forehead, scalp) AK lesion clearance rate data and correlation fits.

The linear correlation fits of AK lesion clearance (1 month and 6 month) versus measured incubated FL for the 15 patients are shown in Fig. 11. Generally, these measurements show the large variability in the patient lesion clearance response related for the measured incubated FL intensity.

The linear fits and corresponding P-values are provided for the patient-specific lesion clearance rates [Figs. 11(a) and 11(b)] and for the site-specific (face, forehead, and scalp) AK clearances [Figs. 11(c) and 11(d)]. The patient-specific clearance showed no statistical significance for both the 1 month [Fig. 11(a)] and the 6 month [Fig. 11(b)] timepoints. The site-specific clearance showed borderline statistical significance for the 1 month timepoint (P=0.066) [Fig. 11(c)], indicating that site-specific clearance (face, scalp, and forehead) should provide better correlation over aggregate patient clearance rates.

**Fig. 12:**
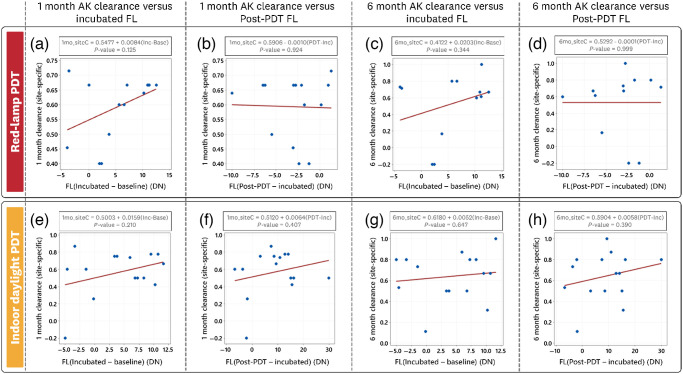
Site-specific AK lesion clearance correlation to measured incubated and photobleached FL intensity for each treatment cohort. The red-lamp treatment AK lesion correlation fits for (a) 1 month clearance versus incubated FL, (b) 1 month clearance versus post-PDT (photobleached) FL, (c) 6 month clearance versus incubated FL, and (d) 6 month clearance versus post-PDT (photobleached) FL. The indoor-daylight treatment AK lesion correlation fits for (e) 1 month clearance versus incubated FL, (f) 1 month clearance versus post-PDT (photobleached) FL, (g) 6 month clearance versus incubated FL, and (h) 6 month clearance versus post-PDT (photobleached) FL.

Linear correlation fits for the cohort (red-lamp and indoor-daylight) AK lesion-specific clearance (1 month and 6 month) to the measured FL intensity (incubated and post-PDT) are provided in Fig. 12.

For the red-lamp PDT cohort [Figs. 12(a)–12(d)], no statistical correlation was found for the measured incubation and post-PDT FL intensity changes. Small positive correlations were observed for the incubated FL (Pearson coefficients <0.3).

For the indoor daylight PDT cohort [Figs. 12(e)–12(h)], no statistical correlation was found for the measured incubation and post-PDT FL intensity changes. Small positive correlations were observed for both the incubated and post-PDT FL (Pearson coefficients <0.3).

#### 3.4.2 AK lesion imaging sample case

**Fig. 13:**
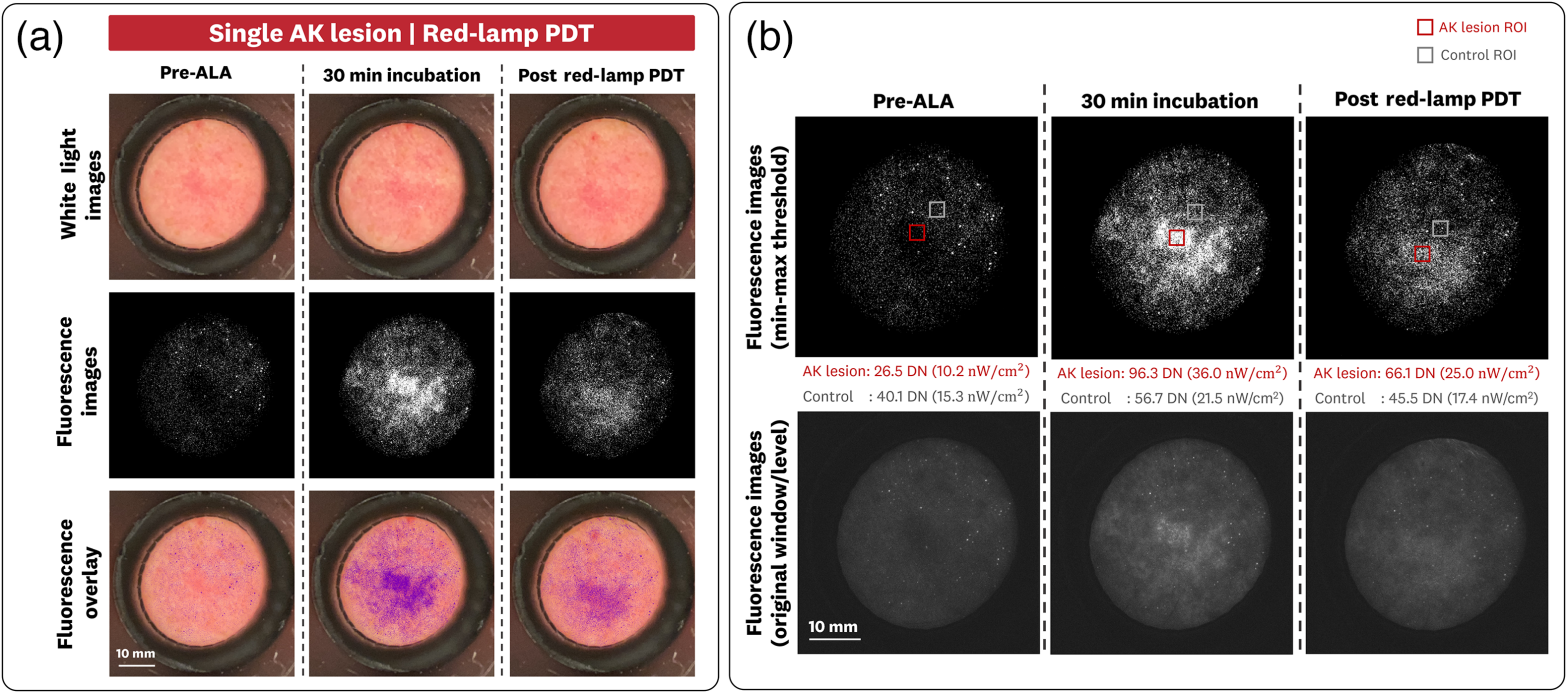
Dual FL and white-light imaging of an individual AK lesion using the 635BP filter configuration. (a) White-light, FL, and FL overlay images showing the increase in FL and photobleaching associated with the PpIX accumulation and red-lamp irradiation, respectively. (b) ROIs of the FL images are taken for the AK lesion and a control region, showing increased levels of FL increase and photobleaching for the AK lesion; FL images that use the original window/level are included to provide further visualization.

The sDUO images of an individual AK lesion (forehead site) are provided in Fig. 13. The white-light images show clear demarcation of the AK lesion. This lesion was treated with red-lamp PDT, such that the FL images showed the expected increase of signal and subsequent photobleaching associated with the PpIX accumulation and red-lamp irradiation, respectively [Fig. 13(a)].

ROIs for the FL images (50×50  pixels, $∼5\;{\mathrm{mm}}^{2}$) were taken for the AK lesion and a control region to determine differences in baseline, incubated, and post-PDT FL [Fig. 13(b)]. For the baseline measurements, the AK and control ROIs measured 26.5±11.9  DN ($10.2\pm 4.7\;\mathrm{nW}/{\mathrm{cm}}^{2}$) and 40.1±13.4  DN ($15.3\pm 5.3\;\mathrm{nW}/{\mathrm{cm}}^{2}$), respectively. This lower FL signal from the AK region might be attributed to the different pigmentation (redness) of the tissue rather than a lower baseline PpIX concentration compared with the control ROI. For the 30-min incubation measurement, the AK and control ROIs measured 96.3±23.95  DN ($36.0\pm 9.3\;\mathrm{nW}/{\mathrm{cm}}^{2}$) and 56.7±16.0  DN ($21.5\pm 6.3\;\mathrm{nW}/{\mathrm{cm}}^{2}$), respectively. The post red-lamp PDT measurement of the AK and control ROIs measured 66.1±17.5  DN ($25.0\pm 6.8\;\mathrm{nW}/{\mathrm{cm}}^{2}$) and 45.5±14.8  DN ($17.3\pm 5.8\;\mathrm{nW}/{\mathrm{cm}}^{2}$), respectively. Using the 1% intralipid + 1% blood phantom data, we estimate the PpIX accumulation and photobleaching for the AK lesion as a 120 nM increase and a 61 nM decrease, respectively. For the control region, we estimate the increase as 25 nM and the decrease as 17 nM. From the radiometric FL measurements and estimated PpIX accumulation, the AK lesion has a >4× accumulation of PpIX compared with the control region.

## 4 Discussion

In this study, we introduced the second iteration of our smartphone-based imager, named sDUO, with the capability to simultaneously acquire radiometric FL and white-light images within the existing PpIX-based PDT clinical workflow.

System characterization of the device enabled the acquisition of reproducible quantitative images using the main imaging sensor’s RAW imaging capabilities alongside fixed imaging parameters. A radiometric calibration of simulated PpIX FL within the optical geometry of the device provided the ability to extrapolate the RAW pixel data (DN) to radiometric units of irradiance ($\mathrm{nW}/{\mathrm{cm}}^{2}$).

The results from the imaged liquid phantoms demonstrated the effect of higher absorber concentration (blood) in reducing the sensitivity of PpIX detection. Furthermore, these tests showed inherent performance differences between the 600LP and 635BP configurations of the device. Although the 600LP filter collects more signal from PpIX emission, the higher throughput of background autofluorescence limits its ability to outperform the 635BP filter.

The liquid phantom measurements provide the best comparison to the sensitivity of previously published systems. Most developed smartphone-based systems for FL imaging have focused on qualitative FL cancer screening, providing no explicit sensitivity characterization and no access to raw imaging for quantitative measurements. ^13^^,^^28^ A published smartphone-based device for imaging PpIX FL for oral cancers showed a lower limit of detection of 1uM in scattering phantoms.^29^ Furthermore, an ultracompact device designed for PpIX imaging showed a lower limit of detection of 10 nM for 1% intralipid solutions.^14^^,^^30^ In comparison with these two devices, the sDUO provided a lower limit of detection of ∼0.5  nM for the 1% intralipid solution, demonstrating over an order of magnitude greater sensitivity than previous devices. This sensitivity is crucial in detecting the low signal generated from the 30 min patient incubation times. A published point probe PpIX dosimeter^6^ showed a lower limit of detection of ∼2  nM for 1% intralipid + 1% blood phantoms. The sDUO provided a lower limit of detection of ∼4  nM for the 1% intralipid + 1% blood phantoms, demonstrating comparable performance to the point-probe dosimeter with the advantages of wide-field measurements, cost-effectiveness and usability within the clinical workflow.

The *in-vivo* murine measurements demonstrated the ability for the sDUO to be used in pre-clinical studies to visualize PpIX accumulation. The heterogeneity of the *in-vivo* measurements highlights the benefit of wide-field FL imaging over point-probe methodologies.

The use of the sDUO within a comparative PDT study^25^^,^^26^ provided insights into differences between the treatment mechanisms of red-lamp and indoor-daylight PDT. For the 15 patients imaged, the sDUO detected the increase in FL signal at the end of the 30 min incubation period for the imaged sites. The red-lamp treatment measurements showed a decreased FL signal caused by PDT photobleaching. The indoor-daylight treatment measurements showed a continued accumulation of FL by the end of the 2 hr irradiation, indicating that the rate of PpIX production surpasses the rate of activation for this treatment modality; additionally this increased signal can be due to local re-synthesis or synthetic redistribution of PpIX.^31^ The results showed that bulk FL measurements from treatment sites (cheek, forehead, and scalp) can be used to detect the changes in FL signal throughout a PDT treatment and determine mechanistic differences between varying treatment regimens. It is worth noting that the provided 30 min measurements are the shortest incubation time reported in the literature for clinical PpIX FL measurements.

Through a combination of the irradiance calibration and the results from the 1% intralipid + 1% blood phantom, the sDUO measurements provided the first *in-vivo* estimation of radiometric FL emission and PpIX concentrations for clinical ALA-PDT patients. The estimated incubated and photobleached concentrations of PpIX fall within previously estimated values in literature.^27^^,^^32^

The linear regression fit of the measured incubated FL to site-specific lesion clearance (face, forehead, and scalp) showed borderline statistical significance with a P-value=0.066. This correlation between the measured FL and treatment outcomes has been reported in both pre-clinical and clinical settings.^6^^,^^33^^–^^35^ Within the clinical setting, the measured photosensitizer FL and erythema have been shown as indicators of treatment response and have been proposed as a way to guide personalized PDT treatment.^15^^,^^35^^,^^36^ The measured heterogeneity in the FL signal and patient clearance rates in this study highlights the challenges of providing personalized PDT guidance at the point-of-care. This FL heterogeneity is further confounded by the erythema induced by the PDT treatment, which affects the detected FL signal due to changing tissue optical properties. Although larger patient cohorts would provide statistical significance for linear regression of measured FL to lesion clearance rates, the clinical relevance of this correlation is limited due to the patient response heterogeneity. This indicates that, although FL measurements can help with understanding clinical results and guiding general treatment regimens, mitigative actions for improving patient outcomes might be best obtained from tracking the clearance rates of previous treatments. This method, using previous patient outcomes as guidance, is viable for PDT because patients require subsequent treatments due to the cumulative nature of the condition as the incidence of AK lesions increases with age.^37^^,^^38^

The presented results provide various use-cases for sDUO imaging in future studies, including (1) the quantification of variability in patient baseline values, incubation, and photobleaching rates; (2) the study of skin pigmentation and erythema on the detected FL emission; (3) the correlation of PpIX FL with AK lesion treatment outcomes; and (4) the exploration of FL surgical guidance for non-melanoma skin cancers.

The main limitations of the sDUO FL measurements are the confounding effects on the measured PpIX signal of skin heterogeneity and the erythema caused through both the incubation and light-therapy processes. The skin heterogeneity introduces variation in the measured FL due to the effects of tissue optical properties, which very among treatment sites and among patients. The AK lesion imaging (Fig. 13) provides a clear example of skin pigmentation effects on the detected FL. The use of a baseline measurement helps to address the site-specific variations but relies on the relative accumulation and photobleaching of the FL signal. The PDT induced erythema further confounds the FL measurement as it provides variation of the tissue optical properties within the same patient site. The ability to correct for tissue optical properties could help to provide absolute PpIX signal measurements rather than relying on the relative signal metrics while addressing the problems that arise with erythema. This correction of optical properties would also account for varying melanin content between patients, which generally lowers the detected FL signal. Phantom studies should be performed to validate the FL signal correction methods, particularly accounting for the layered anatomy of skin optical properties and PpIX accumulation.

Improvements to the device would focus on (1) redesigning components for manufacturability, (2) providing real-time feedback on the adequacy of the image acquisition (i.e., proper contact with patient, room-light bleed-through detection, etc), and (3) generating in-phone FL overlays of the FL and WL images. Further advances could also include the use of a third camera module (available in “Pro” iPhone models) to provide a separate spectral channel or for cross-polarization measurements to highlight superficial skin features. The use of pigmentation maps generated from the white-light images could also be used for correcting FL emission within patients. This correction is the most important step in enabling cross-patient comparison of FL and addressing changes in the FL signal caused by PDT-induced erythema.

The combined liquid phantom, pre-clinical, and clinical measurements indicate that the final design of the system should implement the 635BP filter configuration to maximize the contrast of PpIX accumulation.

## 5 Conclusion

Here, we demonstrated the design and calibration of a dual radiometric FL and white-light imager (sDUO). The phantom, pre-clinical, and clinical measurements demonstrated the capability to provide quantitative measurements of PpIX FL. The sDUO hardware and software design provided realistic deployment within existing PpIX-based PDT clinical workflows, with use within a prospective clinical trial. The system software allowed for the full integration of the RAW image processing pipeline, with the ability to fix image parameters, capture RAW pixel values, and access the image buffer data to report real-time measurements of FL emission. Within the literature, the sDUO provides the first wide-field FL measurements for the 30-min incubation regimens, which contrasts to the 3 hr times used in most studies. Its ability to perform quantitative measurements for these low FL regimes makes it an ideal candidate to be used in future clinical studies. The presented clinical PDT measurements are the first to demonstrate in-vivo mechanistic differences between lamp-based and indoor-daylight treatments. This study is also the first to provide in-vivo radiometric FL measurements and PpIX level estimations for clinical PDT measurements. The sDUO is currently being used in further PDT studies at the Dartmouth-Hitchcock Dermatology, with collaborators at the Massachusetts General Hospital and the Cleveland Clinic using the device to study porphyria and positive margin detection for the resection of basal cell carcinomas.

## Data, Materials, and Code Availability

All relevant data, materials, and software code used in this research are available upon request from the corresponding author.
