# Supplementary material for: Smartphone-based dual radiometric fluorescence and white-light imager for quantification of protoporphyrin IX in skin
Source: J Biomed Opt. 2023 Aug 26;28(8):086003. doi: 10.1117/1.JBO.28.8.086003 (PMC10460113; doi:10.1117/1.JBO.28.8.086003)
Supplement: Supplementary file 1 [file JBO_028_086003_SD001.pdf]

SUPPLEMENTAL MATERIAL

Article Title: Smartphone-based dual radiometric fluorescence and white-light imager for quantification of PpIX in skin

Alberto J. Ruiz,[a,b,*] Richard Allen, Mia K Giallorenzi, Kimberley S. Samkoe,[a] M. Shane Chapman,[b] Brian W. Pogue[a,c]

[a] Thayer School of Engineering, Dartmouth College, Hanover, NH 03755, USA
[b] QUEL Imaging, LLC, White River Junction, VT 0500, USA
[c] Department of Dermatology, Dartmouth Health, Lebanon, NH 03766, USA
[d] Department of Medical Physics, University of Wisconsin-Madison, Madison, WI 53715, USA

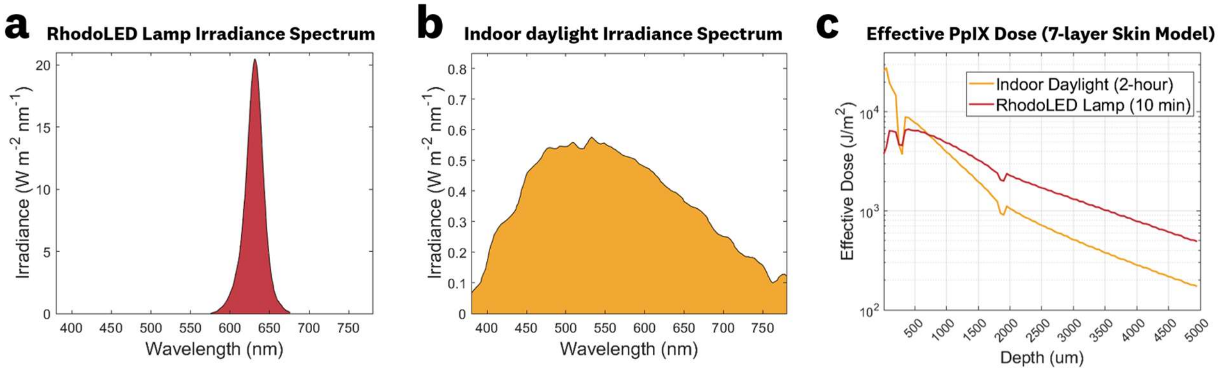

Supplemental Figure 1: Irradiance spectra and equivalent PpIX dose for the RhodoLED Lamp and Indoor-daylight based PDT treatments. A)

Supplementary Table 1: Irradiance, PpIX-effective irradiance, and PpIX-effective fluence for the indoor daylight and RhodoLED lamp based PDT treatments

	Irradiance (W/m^2)	Effective Irradiance (W/m^2)	Effective Fluence (W/m^2) @ DEPTH (um)							
			200	500	750	1000	1250	1500	1750	2000
**Indoor Daylight**	147.8	10.7	2.05	1.08	0.78	0.55	0.39	0.27	0.19	0.15
**RhodoLED**	570.2	9.6	10.4	10.8	9.62	8.09	6.71	5.43	4.23	3.79

Supplementary Table 2: Radiant exposure and effective dose for the RhodoLED lamp (10 min) and indoor daylight (2 h) treatments

	Radiant Exposure (kJ/m^2)	Effective radiant exposure (kJ/m^2)	Effective Dose (kJ/m^2) @ DEPTH (um)							
			200	500	750	1000	1250	1500	1750	2000
**Indoor Daylight**	1064.1	76.7	14,7	7.8	5.6	3.9	2.8	2.0	1.4	1.1
**RhodoLED**	342.1	5.8	6.3	6.5	5.8	4.9	4.0	3.3	2.5	2.3
